# Supplementary figures and images for: Intracellular expression of a fluorogenic DNA aptamer using retron Eco2
Source: eLife. 2026 Mar 3;13:RP99554. doi: 10.7554/eLife.99554 (PMC12956279; doi:10.7554/eLife.99554)

**Ladder  
(nt)**

**130**

**110**

**90**

**70**

**50**

**L  
Eco-2  
Control**

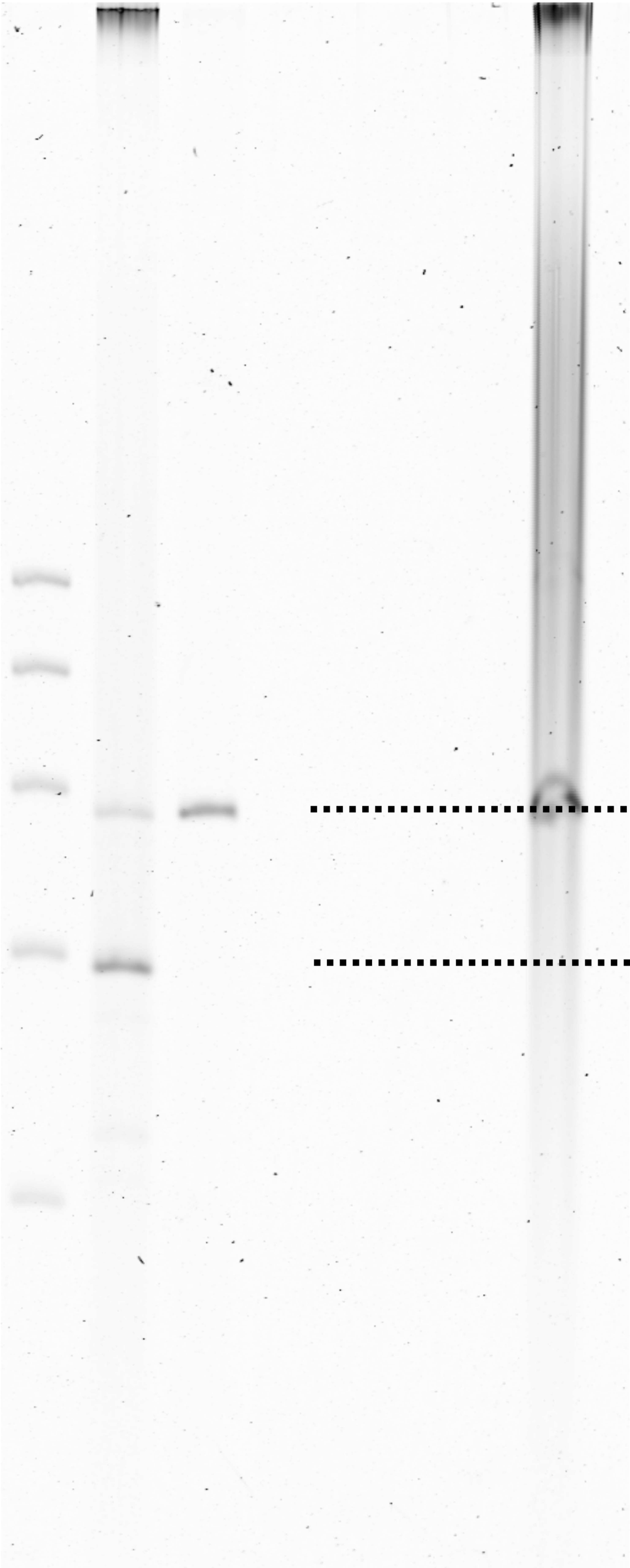

**Eco1**

**Eco2**

Supplement: Figure 2—source data 2. [file elife-99554-fig2-data2.zip › Figure 2 - source data 2/Figure 2 - source data 2.pdf]

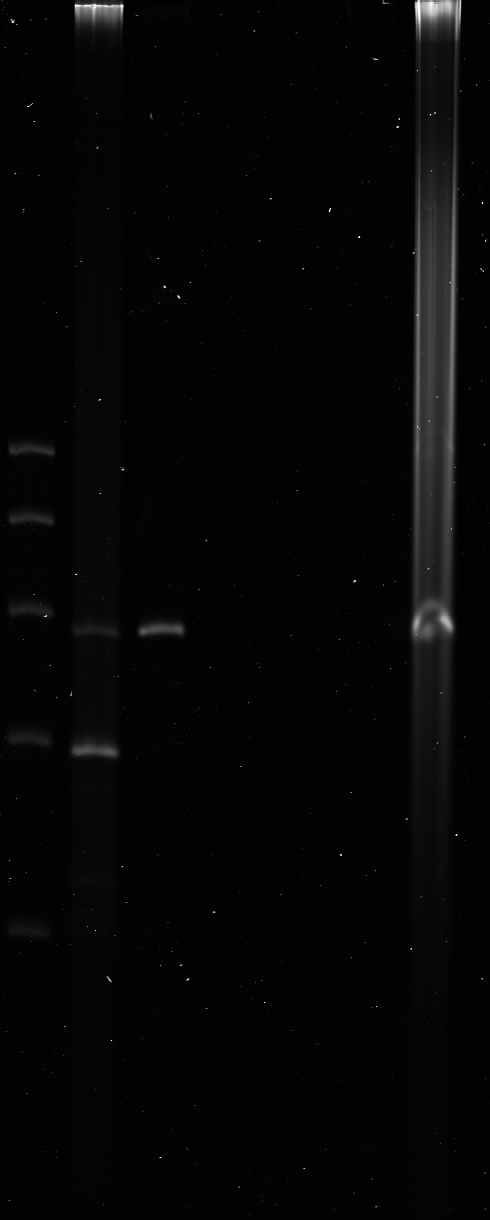

Supplement: Figure 2—source data 3. [file elife-99554-fig2-data3.zip › Figure 2 - source data 3/Figure 2 – source data 3.tif]

L 4L FL 4LE 8LE 11LE FLE  
v1 v2 v3 v4 v1 v2 v3 v4

130 nt

110 nt

90 nt

70 nt

50 nt

30 nt

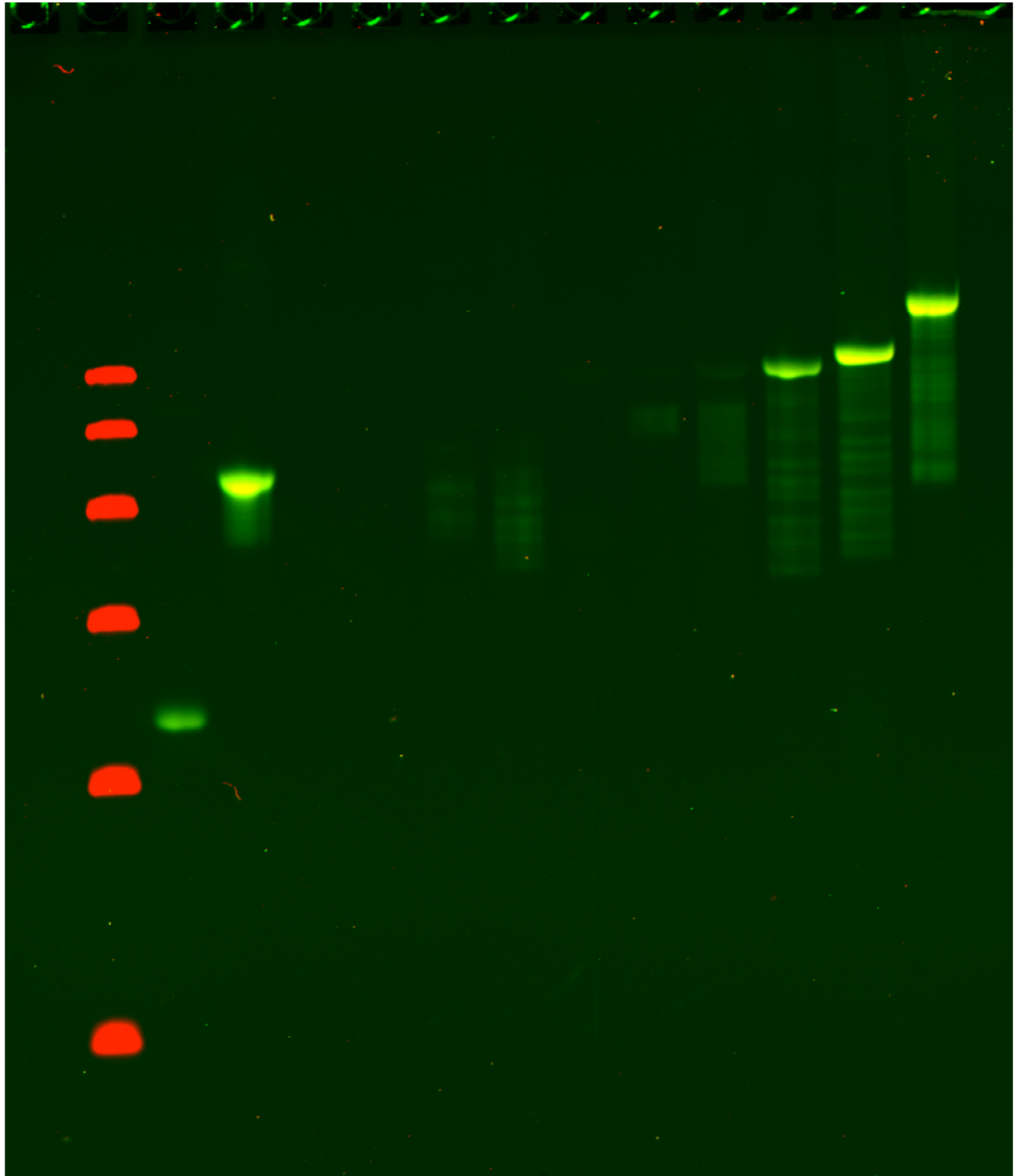

Supplement: Figure 3—source data 1. [file elife-99554-fig3-data1.zip › Figure 3 - source data 1/Figure 3 - source data 1.pdf]

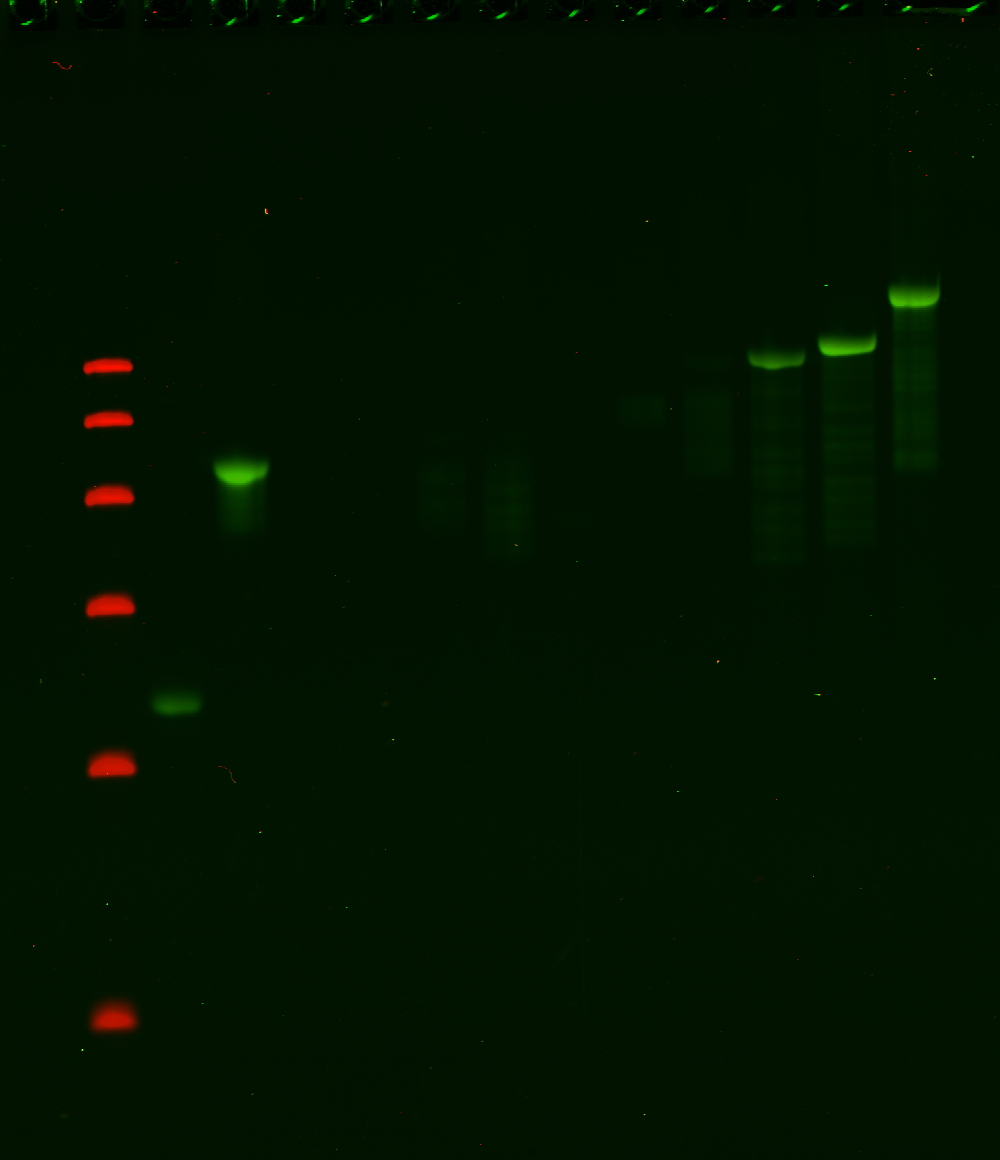

Supplement: Figure 3—source data 2. [file elife-99554-fig3-data2.zip › Figure 3 - source data 2/Figure 3 - source data 2.tif]

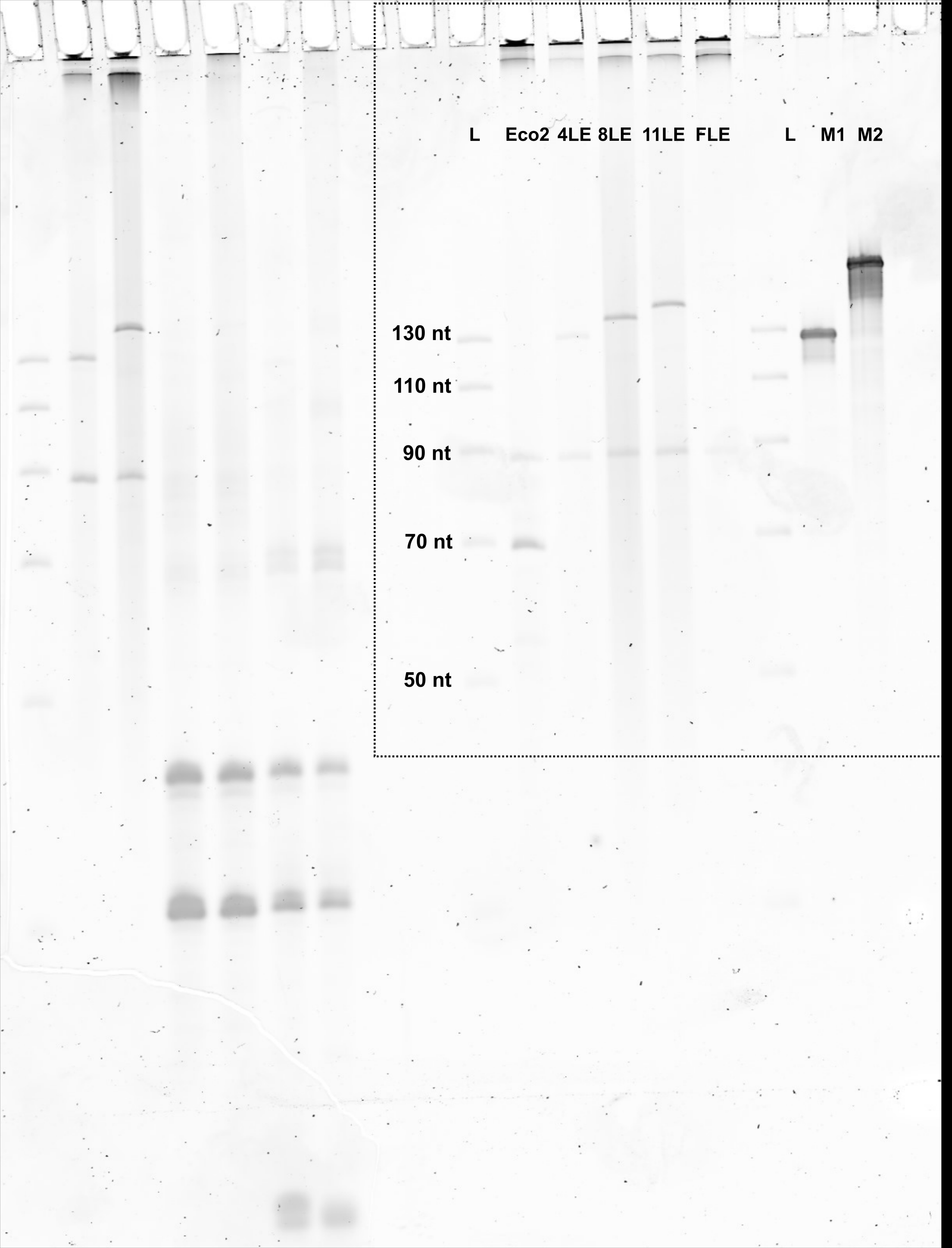

Supplement: Figure 3—source data 3. [file elife-99554-fig3-data3.zip › Figure 3 - source data 3/Figure 3 - source data 3.pdf]

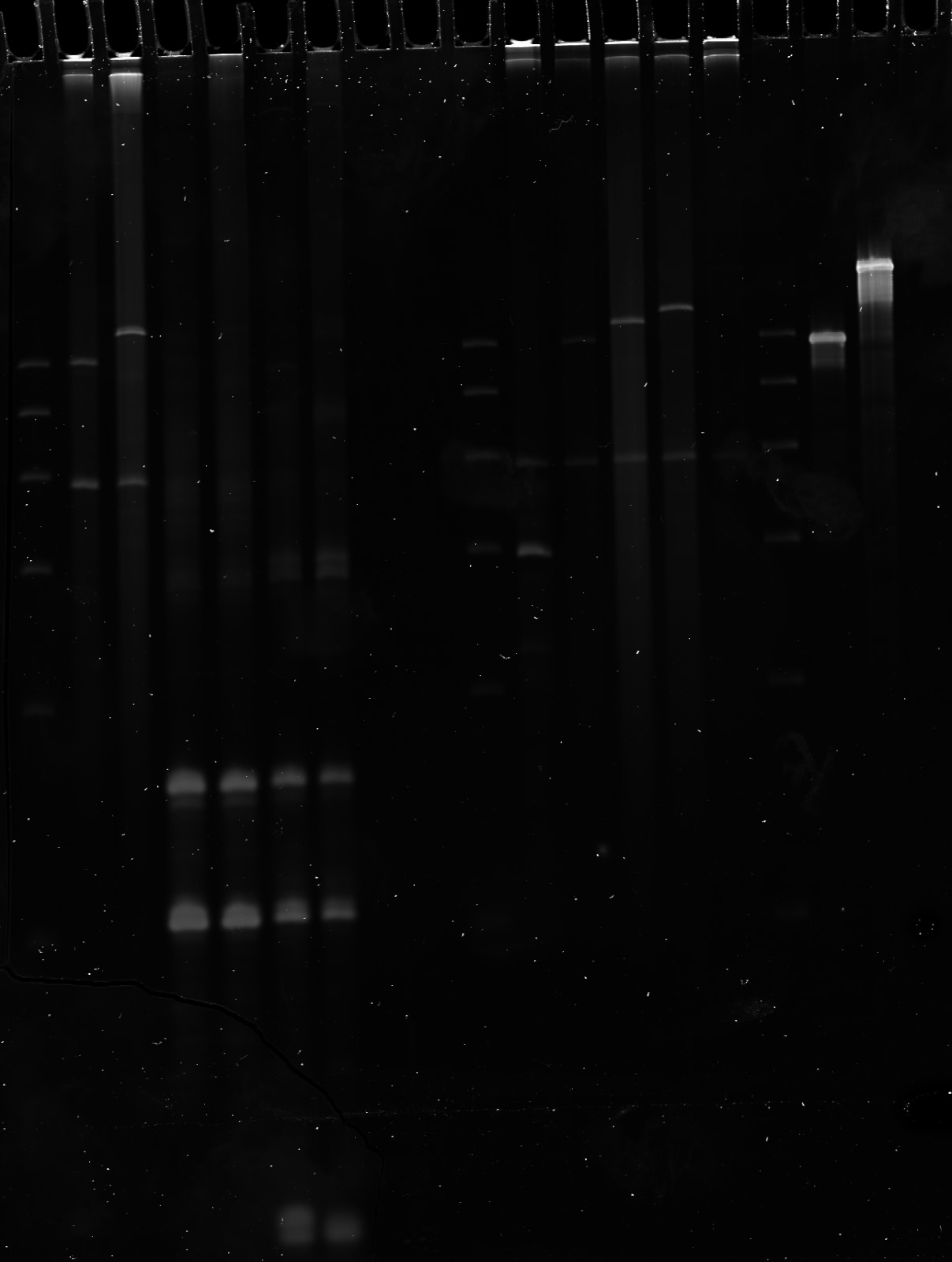

Supplement: Figure 3—source data 4. [file elife-99554-fig3-data4.zip › Figure 3 - source data 4/Figure 3 - source data 4.tif]

**8LEv4 oligo**

**Amount loaded (pmol)**

**L**

**200**

**20**

**10**

**5**

**2**

**1**

**0.2**

**Free lettuce (99 nt)**

**Amount loaded (pmol)**

**200**

**20**

**10**

**5**

**2**

**1**

**0.2**

**L**

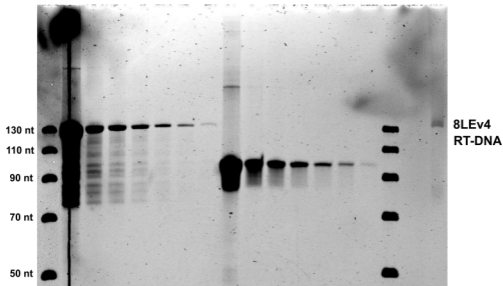

Supplement: Figure 3—source data 5. [file elife-99554-fig3-data5.zip › Figure 3 - source data 5/Figure 3 - source data 5.pdf]

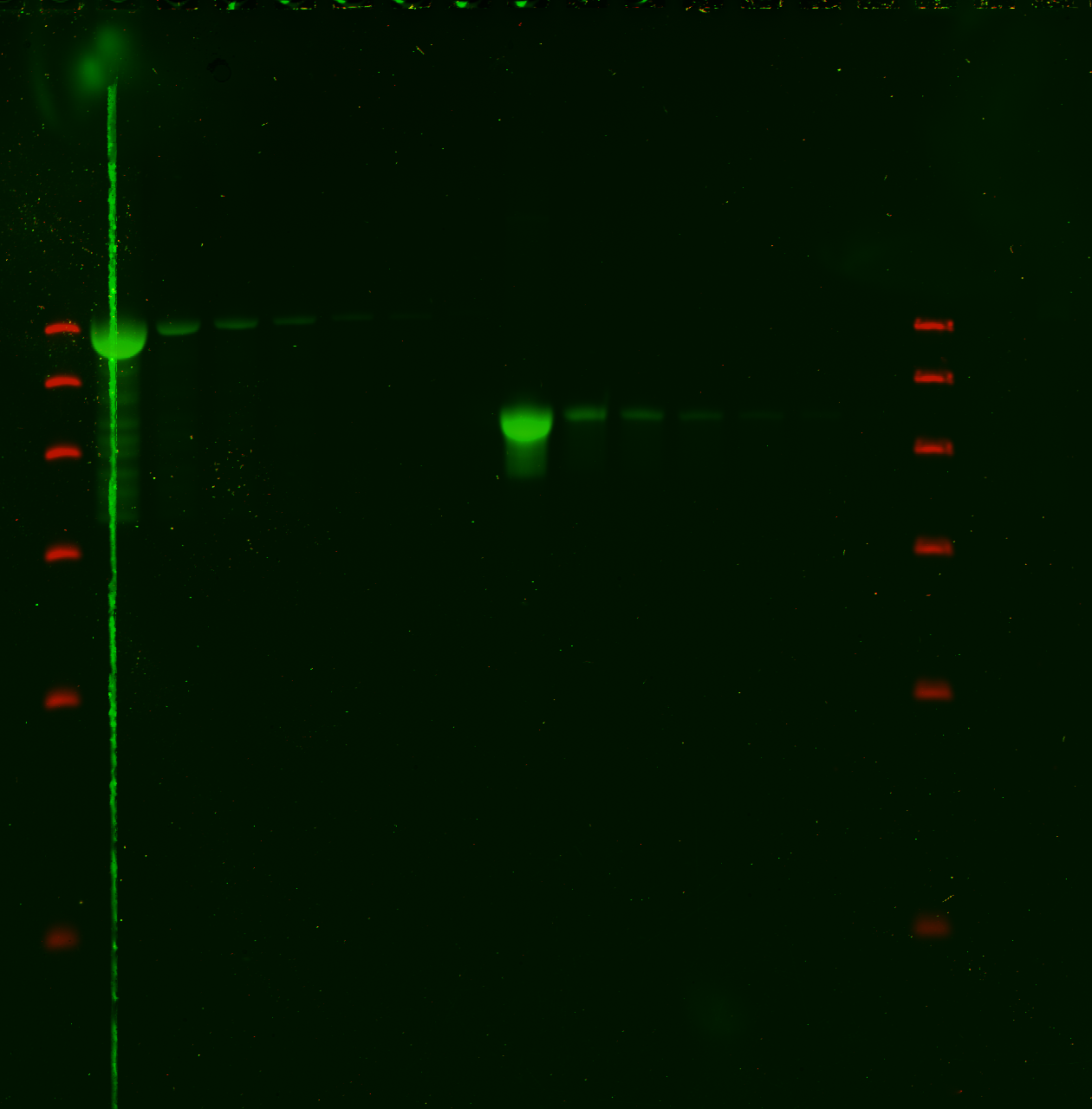

Supplement: Figure 3—source data 6. [file elife-99554-fig3-data6.zip › Figure 3 - source data 6/Figure 3 - source data 6.tif]

**L   Eco2                      4LE                      L   M1**  
**v1   v2   v3   v4**

**130 nt**

**110 nt**

**90 nt**

**70 nt**

**50 nt**

**Eco1**

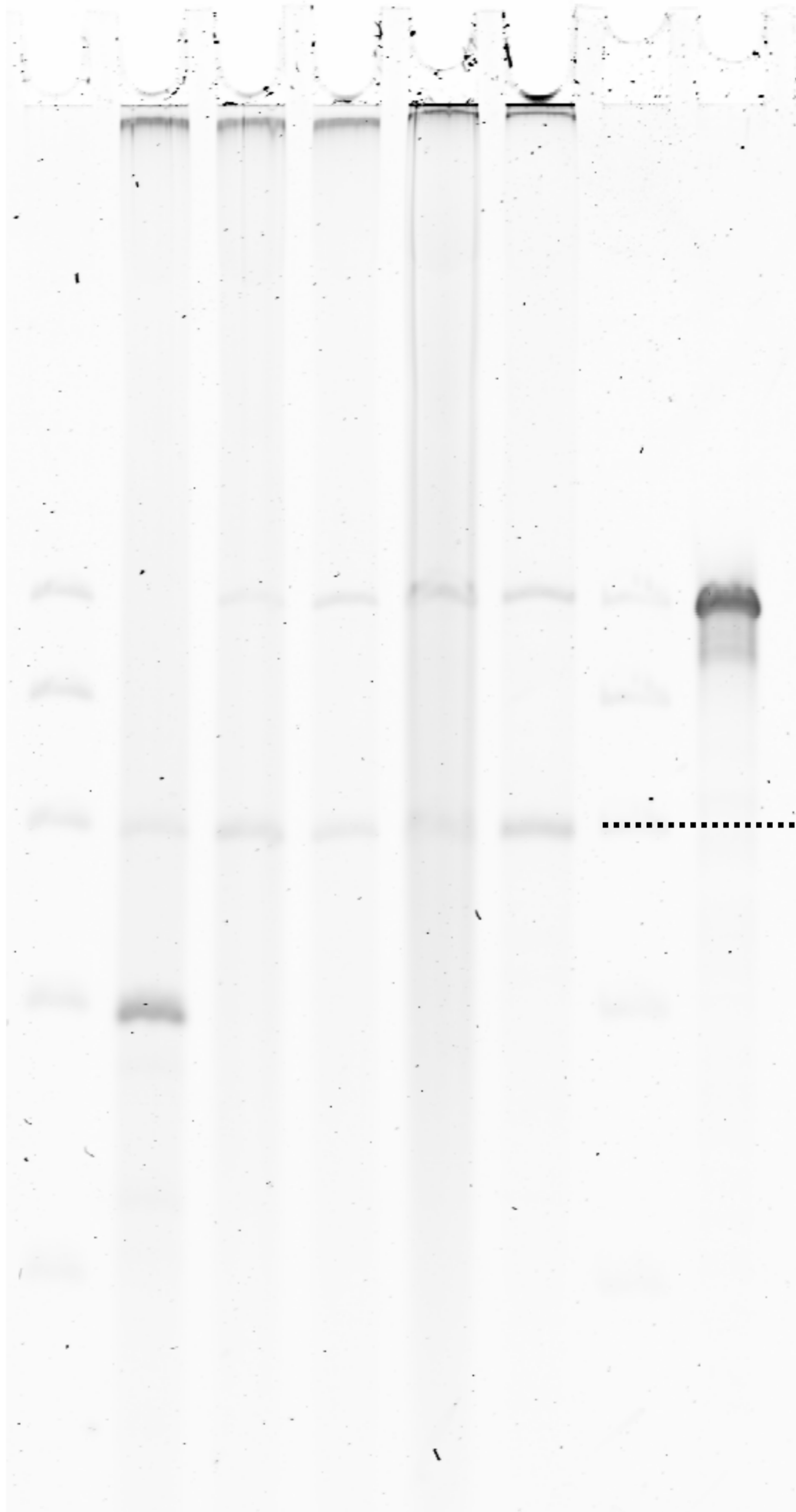

Supplement: Figure 3—figure supplement 1—source data 1. [file elife-99554-fig3-figsupp1-data1.zip › Figure 3 -figure supplement 1 - source data 1/Figure 3 -figure supplement 1 - source data 1.pdf]

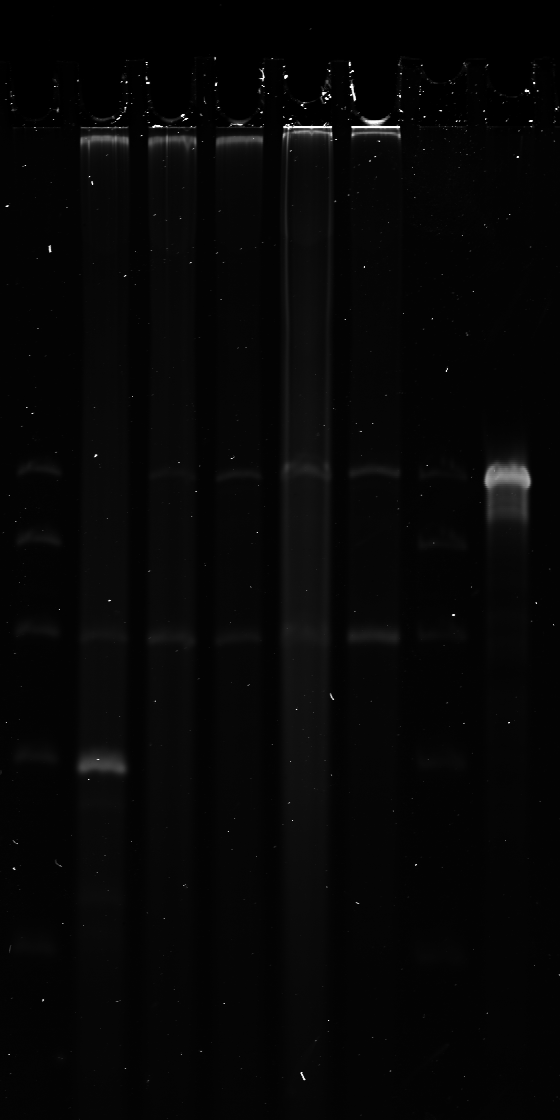

Supplement: Figure 3—figure supplement 1—source data 2. [file elife-99554-fig3-figsupp1-data2.zip › Figure 3 -figure supplement 1 - source data 2/Figure 3 -figure supplement 1 - source data 2.tif]

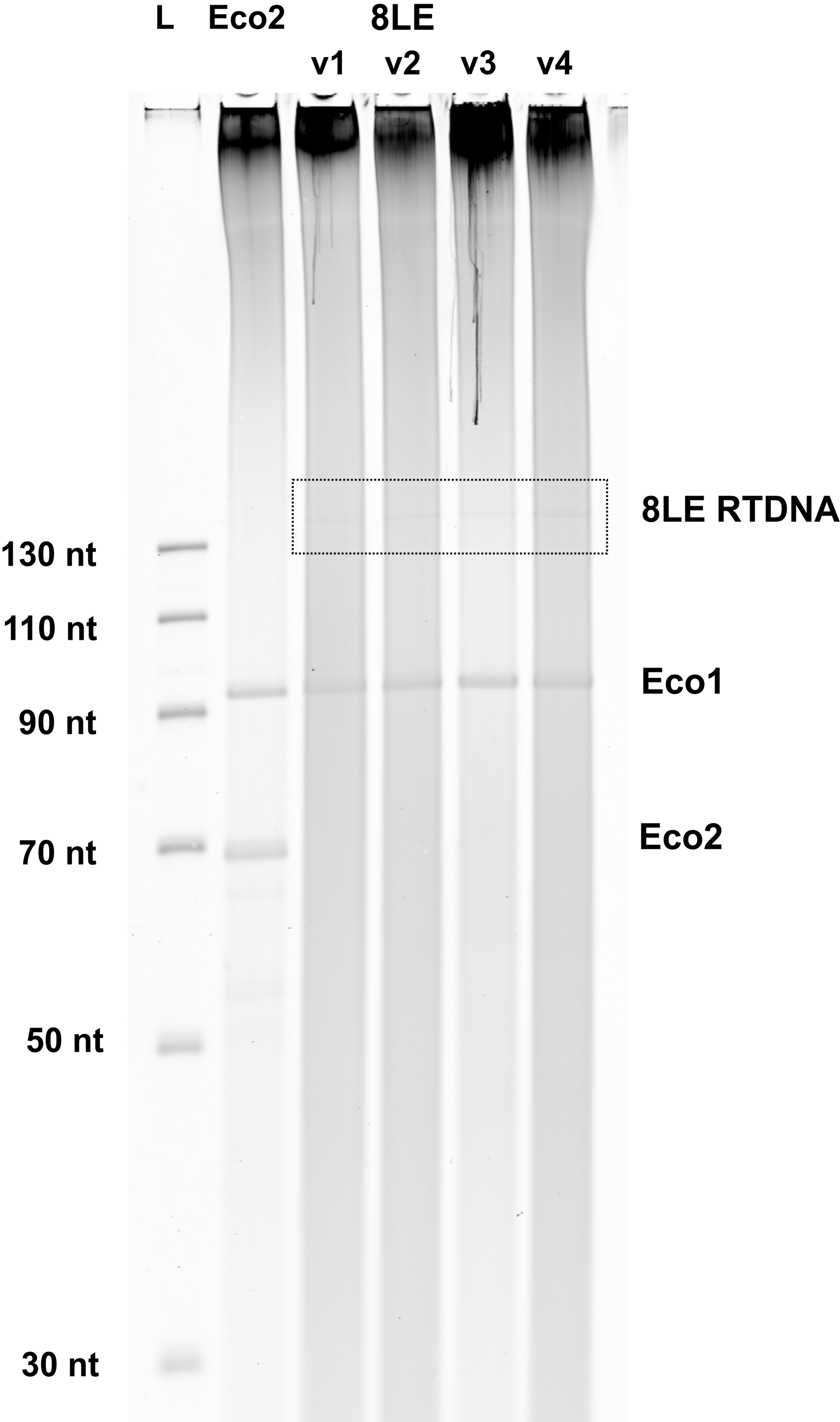

Supplement: Figure 3—figure supplement 3—source data 1. [file elife-99554-fig3-figsupp3-data1.zip › Figure 3 -figure supplement 3 - source data 1/Figure 3 -figure supplement 3 - source data 1.pdf]

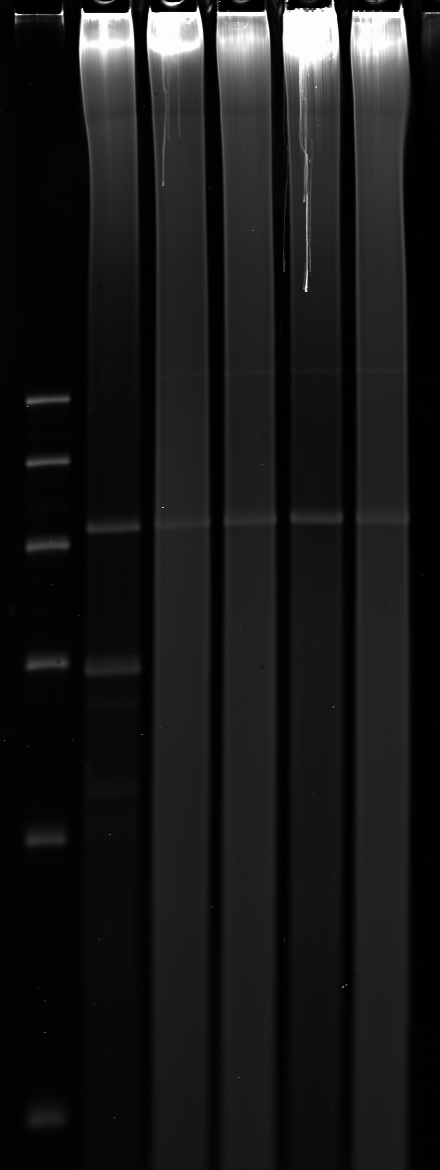

Supplement: Figure 3—figure supplement 3—source data 2. [file elife-99554-fig3-figsupp3-data2.zip › Figure 3 -figure supplement 3 - source data 2/Figure 3 -figure supplement 3 - source data 2.tif]
